# Early risk of acute myocardial infarction following hospitalization for severe influenza infection in the middle-aged population of Hong Kong

**DOI:** 10.1371/journal.pone.0272661

**Published:** 2022-08-09

**Authors:** Ho Yu Cheng, Erik Fung, Kai Chow Choi, Hui Jing Zou, Sek Ying Chair

**Affiliations:** 1 The Nethersole School of Nursing, Faculty of Medicine, The Chinese University of Hong Kong, Hong Kong SAR, China; 2 Laboratory for Heart Failure + Circulation Research, Li Ka Shing Institute of Health Sciences, Prince of Wales Hospital, The Chinese University of Hong Kong, Hong Kong SAR, China; 3 Division of Cardiology and Gerald Choa Cardiac Research Centre, Department of Medicine & Therapeutics, Faculty of Medicine, The Chinese University of Hong Kong, Hong Kong SAR, China; 4 CUHK Medical Centre, The Chinese University of Hong Kong, Hong Kong SAR, China; 5 CARE Programme, Lui Che Woo Institute of Innovative Medicine, CUHK Hong Kong Hub of Paediatric Excellence, Hong Kong Children’s Hospital, Hong Kong SAR, China; 6 Department of Biostatistics and Epidemiology, School of Public Health, Imperial College London, St Mary’s Campus, Norfolk Place, London, United Kingdom; The University of Mississippi Medical Center, UNITED STATES

## Abstract

**Introduction:**

Despite evidence suggesting an association between influenza infection and increased risk of acute myocardial infarction (AMI) in the older adult population (aged 65 years or above), little is known about its near-term risks in middle-aged adults (aged 45 to 64 years). This study aims to estimate the risks of and association between severe influenza infection requiring hospitalization and subsequent AMI within 12 months in middle-aged adults.

**Method:**

This is a retrospective case-control analysis of territorywide registry data of people aged 45 to 64 years admitting from up to 43 public hospitals in Hong Kong during a 20-year period from January 1997 to December 2017. The exposure was defined as severe influenza infection documented as the principal diagnosis using International Classification of Diseases codes and non-exposure as hospitalization for orthopedic surgery. Logistic regression was used to analyze the risk of subsequent hospitalization for AMI within 12 months following the exposure.

**Results:**

Among 30,657 middle-aged adults with an indexed hospitalization, 8,840 (28.8%) had an influenza-associated hospitalization. 81 (0.92%) were subsequently rehospitalized with AMI within 12 months after the indexed hospitalization. Compared with the control group, the risk of subsequent hospitalization for AMI was significantly increased (odds ratio [OR]: 2.54, 95% confidence interval [CI]: 1.64–3.92, p<0.001). The association remained significant even after adjusting for potential confounders (adjusted OR: 1.81, 95% CI: 1.11–2.95, p = 0.02). Patients with a history of hypertension, but not those with diabetes mellitus, dyslipidemia or atrial fibrillation/flutter, were at increased risk (adjusted OR: 5.01, 95% CI: 2.93–8.56, p<0.001).

**Conclusion:**

Subsequent hospitalization for AMI within 12 months following an indexed respiratory hospitalization for severe influenza increased nearly two-fold compared with the non-cardiopulmonary, non-exposure control. Recommendation of influenza vaccination extending to middle-aged adult population may be justified for the small but significant increased near-term risk of AMI.

## Introduction

Influenza is a seasonal communicable disease caused by influenza A or B virus with tropism for the respiratory tract that transmits via direct contact with fomites, self-inoculation and the airborne route [1]. Past influenza pandemics (e.g., 1918 “Spanish flu”, 1958 “Asian flu”, 1968 “Hong Kong flu”) have claimed millions of lives, while recent panzootic epidemics including the highly pathogenic avian influenza H5N1 [2] and a recent outbreak of H5N8 in Russia continue to underscore ongoing risks to vulnerable individuals and the global population, particularly those with cardiovascular and pulmonary disorders.

In spite of early as well as late severe complications from influenza infection that resulted in hospitalizations and deaths [3, 4], its purported epidemiological connection with acute myocardial infarction (AMI) has not been incontrovertible [5–7]. Studies examining the effects of influenza infection on cardiac events found that older adults (aged >65 years) were most vulnerable to poor outcomes including greater risks for complications, hospitalizations, and death [6, 8–10]. The vulnerable cardiovascular period of one year following respiratory hospitalization has been gleaned from the Cardiovascular Health Study and the Atherosclerosis Risk in Communities study cohorts that combined the analysis of middle-aged (45–64 years) adults hospitalized for pneumonia and found a 1.9-fold increased risk for an adverse cardiovascular event [11]. Strong supportive evidence linking serologically confirmed influenza with AMI in the first year after infection came from a recent prospective, multicenter, self-controlled case-series study from Canada that found a 6-fold higher risk for AMI (RR = 6.05, 95% CI: 3.86–9.50, p<0.05) during the first 7 days of influenza infection (risk interval), compared with 52 weeks before and 51 weeks after the risk interval [7]. That study validated previous reports on the associations between prior influenza infection and acute coronary syndrome [6] or heart failure [10]. However, epidemiological data from Asia and the Far East are scarce. A previous study conducted in northern China showed that the risk of AMI was associated with the presence of IgG antibodies to influenza virus A and influenza virus B [12]. Yet, the clear seasonal pattern of influenza in northern China limited the generalizability of study finding in southern China with subtropical climate. Two other studies conducted in Singapore [10] and Hong Kong [6] found associations between influenza surveillance data or aggregated influenza-like illness surveillance data and MI-associated hospitalization and deaths with adjustment for seasonality and relevant environment confounders. This is the first study that adopted an individual patient cohort analysis of territorywide registry data of people aged 45 to 64 years who were admitted into public hospitals in Hong Kong during a 20-year period.

The Centers of Disease Control and Prevention indicated that persons aged 50 years or above belong to the priority groups for seasonal influenza vaccination because they are at higher risk for medical complications attributable to severe influenza than general population [13]. Previous studies showed that free influenza vaccination increased the vaccination rate [14, 15]. However, countries/region with free or subsidized vaccination policy, including Australia [16], New Zealand [17], Singapore [18] and Hong Kong [19], do not include middle-aged (age 45 to 64 years old) adults as eligible recipients. As Hong Kong is an epidemiological hotspot for influenza epidemics in Southern China, a detailed review and characterization of regional health records, in addition to the use of individual patient cohort analysis, may provide insight into disease patterns and inform public health strategy development. In this 20-year retrospective individual patients cohort analysis of uninterrupted electronic health records from a public healthcare system that served over 90% of the Hong Kong population, we aimed to determine the extent to which prior influenza-associated hospitalization contributed to subsequent hospital admission for AMI within 12 months in middle-aged adults.

## Materials and methods

### Data sources

An electronic health record and clinical database system operated by the Hospital Authority that served up to 43 Hong Kong public hospitals was used to query discharge diagnoses and outcomes based on International Classification of Disease, Ninth Revision (ICD-9), codes. The relevant socio-demographic and clinical data were compiled on individuals aged 45–64 years (defined as ‘middle-aged’) who had been hospitalized during the period between January 1, 1997 and December 31, 2017. To protect patient privacy, the Hospital Authority had assigned an investigator-nonidentifiable unique alias code number to each individual before releasing the data file for this research. This study was approved by the Survey and Behavioral Research Ethics Committee of the Chinese University of Hong Kong with the wavier of informed consent because the data was deidentified.

### Exposed and non-exposed groups

The exposed group included middle-aged adults who were hospitalized between January 1997 and December 2017 with the principal diagnosis of influenza (ICD-9 codes 487, 487.0, 487.1, 487.8, and 488) that constituted the indexed hospitalization. The non-exposed comparator group comprised middle-aged adults without a cardiopulmonary indication for hospitalization who were admitted with the principal diagnosis of severe orthopedic problems, including fracture of the neck of femur, fracture of the humerus, and fracture of tibia and fibula, that required surgical intervention. Patients admitted for orthopedic surgery were included in the non-exposed group as their conditions were usually independent of influenza or related problems.

### Subsequent hospitalization for new-onset AMI within 12 months

Patients’ information related to hospitalization for AMI in the first 12 months following index hospitalization was retrieved. The outcome of subsequent hospitalization for AMI was considered as positive if the patient had a principal diagnosis of new-onset AMI based on the ICD-9 code 410.

### Data analysis

Data were summarized and presented using appropriate descriptive statistics. Length of the indexed hospital stay was square root-transformed before subject to inferential analysis. Subsequent hospitalization for AMI within one year after discharge from the indexed hospitalization, the outcome of the study, was coded as a binary variable (Yes/No). To avoid computational convergence problems, logistic regression was used, instead of log-binomial regression, to estimate the risk ratio of subsequent one-year hospitalization for AMI of the exposed group (influenza-associated hospitalization) versus the non-exposed group (non-cardiopulmonary disease-associated hospitalization). Unadjusted and adjusted logistic regression analyses were conducted with adjustment for demographics and medical conditions in the clinical history. Odds ratios (ORs), which yielded a good approximation of the risk ratio of a rare outcome, were calculated. Specifically, a hierarchical approach was used for the adjusted analyses using the following batches of potential confounders successively included in the adjusted models: (a) age, sex, length of the indexed hospital stay, and medical history of hypertension, diabetes mellitus, dyslipidemia, and atrial fibrillation or atrial flutter, and (b) whether or not the indexed hospitalization occurred after the H1N1 pandemic (April 2009) or in winter (November to March) and non-winter (April to October) seasons. We did not consider the time effect in the adjusted models because there was no notable trend or cycle in the incidence of subsequent hospitalization for AMI over the 20-year observation period. All the statistical analyses were performed using SAS 9.4 (SAS Institute Inc., Cary, NC, USA) and all tests were 2-sided at a 5% level of significance.

## Results

### Patient characteristics

Of 30,657 middle-aged adults hospitalized during the study period, 8,840 (28.8%, mean age 56.4 years) had influenza and 21,817 (61.2%; mean age, 56.3 years) identified as the non-exposed control had undergone orthopedic surgery. The median length of hospital stay was 3 and 7 days for the influenza and the control group, respectively. The control group had fewer cardiovascular comorbidities including hypertension, diabetes, dyslipidemia, and atrial fibrillation or atrial flutter compared with the influenza group (Table 1).

**Table 1:** Characteristics of the study population (N = 30,657).

	No. (%)			
		Influenza-associated hospitalization		
Characteristics	All (N = 30,657)	No (n = 21,817)	Yes (n = 8,840)	P-value
Age, mean (SD)	56.3 (6.0)	56.3 (6.0)	56.4 (5.8)	0.283^a^
Sex				
Female	15548 (50.7)	11032 (50.6)	4516 (51.1)	0.409^b^
Male	15109 (49.3)	10785 (49.4)	4324 (48.9)	
Length of the index hospital stay, days, median (interquartile range)	5 (2–11)	7 (3–14)	3 (2–4)	<0.001^c^*
History of hypertension				
No	27233 (88.8)	19886 (91.1)	7347 (83.1)	<0.001^b^*
Yes	3424 (11.2)	1931 (8.9)	1493 (16.9)	
History of diabetes mellitus				
No	27462 (89.6)	19839 (90.9)	7623 (86.2)	<0.001^b^*
Yes	3195 (10.4)	1978 (9.1)	1217 (13.8)	
History of dyslipidemia				
No	29390 (95.9)	21133 (96.9)	8257 (93.4)	<0.001^b^*
Yes	1267 (4.1)	684 (3.1)	583 (6.6)	
History of atrial fibrillation or atrial flutter				
No	30045 (98.0)	21554 (98.8)	8491 (96.1)	<0.001^b^*
Yes	612 (2.0)	263 (1.2)	349 (3.9)	
Index hospital admission period				
Before April 2009	12557 (41.0)	11244 (51.5)	1313 (14.9)	<0.001^b^*
On or after April 2009	18100 (59.0)	10573 (48.5)	7527 (85.1)	
Index hospital admission in winter and summary				
No	17438 (56.9)	12378 (56.7)	5060 (57.2)	0.419^b^
Yes	13219 (43.1)	9439 (43.3)	3780 (42.8)	

^a^ Independent t-test;

^b^ chi-square test;

^c^ square root-transformed before subjected to independent t-test.

*p<0.05.

### Risk of subsequent AMI hospitalization within 12 months after influenza-associated hospitalization

Within 12 months after the index respiratory hospitalization, a total of 81 (0.92%) patients were admitted to the hospital with new-onset AMI. Among those hospitalized for AMI, there was no significant difference in the length of the index hospitalization (OR: 1.03, 95% CI: 0.92–1.14, p = 0.66) compared to those without subsequent AMI hospitalization. The incidence rate of subsequent 12-month AMI hospitalization was significantly increased in older male patients with a history hypertension, diabetes mellitus, and dyslipidemia (Table 2). The incidence of subsequent AMI hospitalization within 12 months was significantly higher in patients with previous influenza-associated hospitalization compared with those without (0.5% versus 0.2%).

**Table 2:** Characteristics of the study cohort by status of subsequent hospitalization for acute myocardial infarction within 12 months after discharge from index hospitalization.

	No. (%)			
	Subsequent hospitalization for myocardial infarction			
Characteristics	No (n = 30,576)	Yes (n = 81)	OR (95% CI)	P-value
Age, mean (SD) ^†^	56.3 (6.0)	59.9 (4.8)	3.24 (2.06–5.08)	<0.001*
Sex				
Female	15523 (99.8)	25 (0.2)	1	
Male	15053 (99.6)	56 (0.4)	2.31 (1.44–3.70)	<0.001*
Length of indexed hospital stay, days, median (interquartile range) ^‡^	5 (2–11)	6 (3–11)	1.03 (0.92–1.14)	0.657
History of hypertension				
No	27196 (99.9)	37 (0.1)	1	
Yes	3380 (98.7)	44 (1.3)	9.57 (6.17–14.84)	**<**0.001*
History of diabetes mellitus				
No	27412 (99.8)	50 (0.2)	1	
Yes	3164 (99.0)	31 (1.0)	5.37 (3.43–8.42)	**<**0.001*
History of dyslipidemia				
No	29327 (99.8)	63 (0.2)	1	
Yes	1249 (98.6)	18 (1.4)	6.71 (3.96–11.36)	**<**0.001*
History of atrial fibrillation or atrial flutter				
No	29966 (99.7)	79 (0.3)	1	
Yes	610 (99.7)	2 (0.3)	1.24 (0.31–5.07)	0.761
Index hospital admission period				
Before April 2009	12539 (99.9%)	18 (0.1%)	1	
On or after April 2009	18037 (99.7%)	63 (0.3%)	2.43 (1.44–4.11)	**<**0.001*
Index hospital admission in winter and summer				
No	17387 (99.7%)	51 (0.3%)	1	
Yes	13189 (99.8%)	30 (0.2%)	0.78 (0.49–1.22)	0.270
Influenza-associated hospitalization				
No	21777 (99.8%)	40 (0.2%)	1	
Yes	8799 (99.5%)	41 (0.5%)	2.54 (1.64–3.92)	**<**0.001*

OR, odds ratio; CI, confidence interval.

^†^ORs were estimated per 10-year increment.

^‡^Length of the indexed hospital stay was presented as median (interquartile range) and square root-transformed in estimating the OR.

*p<0.05.

As shown in Table 3, the overall risk of subsequent AMI hospitalization within 12 months following the index influenza-associated hospitalization was significantly higher than that of orthopedic-associated hospitalization. Adjustment for index hospitalization occurring after the 2009 H1N1 pandemic (April 2009) (adjusted OR: 1.46, 95% CI 0.83–2.58, p = 0.19) or by season (winter [November to March] and non-winter [April to October]) (adjusted OR: 0.74, 95% CI 0.47–1.17, p = 0.20) did not significantly affect the risk estimate. In the fully adjusted logistic regression model, the risk was estimated at adjusted OR 1.81(95% CI 1.11–2.95, p = 0.02). Of note, hypertension (adjusted OR 5.01, 95% CI 2.93–8.56, p<0.001), but not dyslipidemia (adjusted OR: 1.64, 95% CI 0.91–2.95, p = 0.13), diabetes mellitus (adjusted OR: 1.55, 95% CI 0.91–2.64, p = 0.10) or atrial fibrillation/flutter (adjusted OR 0.40, 95% CI 0.10–1.65, p = 0.22), was an independent predictor of rehospitalization for AMI. The significant higher risk of subsequent AMI hospitalization following index influenza-associated hospitalization compared with that of orthopedic-associated hospitalization was found within 6 months (adjusted OR: 2.45, 95% CI:1.32–4.53, p = 0.004) and 12 months (adjusted OR: 1.81, 95%CI: 1.11–2.95, p = 0.02) (Table 4).

**Table 3:** Results from logistic regression analysis of subsequent hospitalization for acute myocardial infarction within 12 months following discharge from hospital for severe influenza (exposed), compared with elective orthopedic surgery (non-exposed).

	Model 1		Model 2		Model 3	
	OR (95% CI)	P-value	OR (95% CI)	P-value	OR (95% CI)	P-value
Influenza-associated hospitalization	2.54 (1.64–3.92)	<0.001*	2.01 (1.25–3.22)	0.004*	1.81 (1.11–2.95)	0.017
Age (per 10-year increment)			2.52 (1.59–4.00)	<0.001*	2.50 (1.57–3.97)	<0.001*
Male			2.40 (1.49–3.85)	<0.001*	2.39 (1.48–3.84)	<0.001*
Length of indexed hospital stay (days) ^‡^			1.05 (0.94–1.17)	0.395	1.06 (0.96–1.17)	0.288
History of hypertension			5.01 (2.93–8.56)	<0.001*	4.88 (2.86–8.35)	<0.001*
History of diabetes			1.55 (0.91–2.64)	0.108	1.56 (0.92–2.65)	0.103
History of dyslipidemia			1.64 (0.91–2.95)	0.102	1.58 (0.88–2.86)	0.129
History of atrial fibrillation or atrial flutter			0.40 (0.10–1.65)	0.205	0.41 (0.10–1.69)	0.217
Index hospital admission period on or after April 2009					1.46 (0.83–2.58)	0.190
Index hospital admission in winter and non-winter seasons					0.74 (0.47–1.17)	0.200

OR, odds ratio; CI, confidence interval.

^‡^*Length of the indexed hospital stay was square root-transformed in estimating the OR. *p<0.05Model 1: unadjusted model with only type of indexed hospitalization entered.

Model 2: adjusted for age, sex, medical history of hypertension, diabetes, dyslipidaemia, and atrial fibrillation/atrial flutter.

Model 3: adjusted for covariates in Model 2 + period (before or after April 2009) and season (winter: November to March; non-winter: April to October).

**Table 4:** Results from logistic regression analyses of subsequent hospitalization for acute myocardial infarction within 1, 3, 6 and 12 months following discharge from hospital for severe influenza (exposed), compared with elective orthopedic surgery (non-exposed).

Subsequent hospitalization for myocardial infarction	Unadjusted model		Adjusted model^†^	
*Risk intervals*	OR (95% CI)	P-value	OR (95% CI)	P-value
Within 1 month (n = 19)	2.22 (0.90–5.47)	0.082	1.34 (0.50–3.61)	0.565
Within 3 months (n = 32)	2.80 (1.40–5.61)	0.004*	1.95 (0.91–4.17)	0.085
Within 6 months (n = 51)	3.26 (1.87–5.68)	<0.001*	2.45 (1.32–4.53)	0.004*
Within 12 months (n = 81)	2.54 (1.64–3.92)	<0.001*	1.81 (1.11–2.95)	0.017*

OR, odds ratio; CI, confidence interval.

^†^With adjustment for age, sex, medical history of hypertension, diabetes, dyslipidaemia, and atrial fibrillation/atrial flutter, period (before or after April 2009) and season (winter: November to March; non-winter: April to October).

*p<0.05.

## Discussion

This study has leveraged the availability of a 20-year territorywide data set from the Hong Kong public healthcare system to examine the association between prior influenza infection and subsequent new-onset AMI hospitalization in middle-aged adults. The incidence of subsequent hospitalization for AMI in the first year after index hospitalization increased nearly two-fold in the exposed compared with the non-exposed control (i.e., patients hospitalized for orthopedic surgery). Our findings suggest that influenza infection can heighten the subsequent 12-month risk of AMI in a small (0.92%) but significant proportion of middle-aged adults.

Our results concur with previous studies reporting increased hospitalization for AMI during the seasonal influenza outbreak period [8] and an increased risk of AMI following influenza infection [7, 20, 21]. In a time-series analysis by Nguyen et al., it was determined that each interquartile increase in influenza incidence was associated with an increase of between 5.8% (95% CI: 2.5%–9.1%) and 13.1% (95% CI: 5.3%–20.9%) for AMI mortality in the following 14 days [21]. Furthermore, patients with AMI and coexisting influenza infection had greater risks for adverse outcomes than those with AMI but no influenza infection [22, 23]. A study of 4,285,641 patients with AMI found that coexisting influenza was associated with significantly higher risks for in-hospital mortality (OR: 1.26, 95% CI: 1.05–1.50, p = 0.01), acute kidney injury (OR: 1.36, 95% CI: 1.21–1.54, p<0.01), acute kidney injury requiring dialysis (OR: 1.92, 95% CI: 1.33–2.78, p<0.001), multi-organ failure (OR: 1.81, 95% CI: 1.59–2.07,p<0.01), and increased length-of-stay (7 days vs 5 days), compared to those without coexisting influenza [22].

Previous studies have reported an epidemiological link between influenza infection and cardiovascular events predominantly in the older adult populations [6, 7, 10]. In this study, we found a two-fold increase in the risk of hospitalization for AMI in the subsequent 12 months after influenza-associated hospitalization among middle-aged people, in line with previous studies [11, 24]. A study from Hong Kong by Wong et al. reported that laboratory-confirmed influenza was significantly associated with an excess hospitalization at a rate of 5.3 per 1,000,000 population (95% CI: 0.5–9.5) for coronary heart disease in people aged 40–64 years [24]. Collectively, our study and those by others suggested that the long-term risk for AMI after influenza infection is not insignificant in middle-aged adults.

While the viral pathogenic mechanisms remain poorly understood, Muscente and De Caterina recently provided a comprehensive review summarizing the multiple pathways including a heightened inflammatory response, altered vasomotor tone, endothelial dysfunction, platelet aggregation, and thrombus formation that could predispose to acute coronary syndrome and atherothrombosis in patients with atherosclerotic cardiovascular disease [25]. Although the exact pathogenic mechanisms underlying influenza infection and its associated increased risk for AMI remain largely unclear, the proinflammatory milieu is culpable [25, 26]. Animal studies have shown a direct role for influenza in infecting and promoting vascular inflammation in the co-presence of macrophage infiltration of the arterial wall [27]. Clinical and epidemiological evidence for increased vascular inflammation and thrombogenicity with influenza infection adversely affecting atherosclerotic plaque stability was seen in studies demonstrating seasonal infections during winter season in association with increased procoagulant factors and/or activity and increased deaths from cardiovascular disease [28] and a reduced incidence of AMI with influenza vaccination [29].

Influenza vaccination is the primary strategy to reduce the lethality and morbidity of influenza infection [30]. Influenza vaccination may reduce subsequent risk for AMI or cardiovascular disease by 16% to 45% [31–33]. A population-based case-control study found that influenza vaccination could reduce the risk for AMI by 20% (adjusted OR: 0.80, 95% CI: 0.76–0.84, p<0.01) [32]. A meta-analysis of self-controlled case-series also showed a 16% lower risk of myocardial infarction (incident rate ratio: 0.84, 95% CI: 0.78–0.91) in the first four weeks following the influenza vaccination [20]. While the Centres for Disease Control Prevention recommends influenza vaccination for people aged over 50 years to reduce morbidity [13], our findings support extending this recommendation to middle-aged adults, especially those with hypertension, to mitigate AMI risks.

The use of an unabridged, 20-year territory-wide data set retrieved from an electronic health record and clinical database system is a major strength of this study. However, there are limitations including the possibility of unmeasured factors, or latent variables, that could affect our estimates despite best efforts in adjusting for potential confounders. Moreover, a variety of clinical and laboratory tests values and data were not accessible or available, including influenza virus type and serotype, uptake of influenza vaccines, and longitudinal inventory of medications, among others. Other environmental (e.g., living condition, type of housing, air population index, ambient temperature, humidity) factors that were not studied could affect the accuracy of interpretations, since both influenza epidemic and AMI exhibit seasonal patterns that may be modulated by the environment. Nevertheless, we have adjusted for seasonal effects (i.e., winter or non-winter) and other demographic and clinical factors, where appropriate. Another study limitation is our focus on an inpatient population with severe influenza infections that likely represented a minority of the territory’s population, as most infected individuals probably had mild or no symptoms and were not admitted into hospitals [4]. The findings on influenza-associated hospitalization in this study likely represented severe cases. While influenza-associated hospitalization and the subsequent AMI hospitalization impose a significant burden among middle-aged adults in subtropical area, future study is suggested to examine the cost benefit of influenza vaccination by comparing the cost of vaccine and general practitioner consultation with the savings from influenza-associated hospitalization.

## Conclusion

This territorywide study adds to a growing body of evidence that severe influenza infection requiring hospitalization increases subsequent risks for future AMI by nearly two-fold in middle-aged adults, particularly in patients with preexisting hypertension.
